# Case Report: A rare cause of duodenal intussusception: the pedunculated Brunner’s gland hamartoma

**DOI:** 10.3389/fonc.2025.1699786

**Published:** 2025-10-08

**Authors:** Yi-Hua Wang, Jing Zhou, Xiao-Shan Huang, Xiao-Zhong Zheng, Meng-Ying Gu, Jia-Qi Duan, Shu-Feng Fan, Jian-Xia Xu

**Affiliations:** ^1^ Department of Radiology, The Second Affiliated Hospital of Zhejiang Chinese Medical University, Hangzhou, Zhejiang, China; ^2^ Department of Radiology, Affiliated Hospital of Hangzhou Normal University, Hangzhou, Zhejiang, China

**Keywords:** intestinal polyps, Brunner’s gland adenoma, Brunner’s gland hamartoma, computed tomography, endoscopic therapy

## Abstract

**Background:**

Brunner’s gland hamartomas (BGHs) are primarily benign and typically do not exhibit characteristic clinical symptoms. The discovery and diagnosis of duodenal BGHs may require imaging assistance. Dissemination of this case can help inform future diagnosis and effective treatment.

**Case summary:**

Herein, we report a case of a 43-year-old man presenting with bloating. The computed tomography (CT) found a strip-like, mixed-density lesion with fat attenuation in the duodenal canal, and the proximal intestine was dilated. Contrast-enhanced abdominal CT revealed that the lesion had mild to moderate enhancement with incomplete intussusception. The lesion was resected entirely with the endoscope. Postoperative pathology results confirmed a BGH.

**Conclusion:**

CT findings exhibit specific characteristics that are valuable for visualizing these lesions or their secondary manifestations. An elongated intraluminal mass lesion with heterogeneous density and fat attenuation may indicate a pedunculated duodenal Brunner’s gland hamartoma.

## Introduction

The duodenal Brunner’s gland hamartoma (BGH), also known as Brunner’s gland adenoma (BGA), is typically benign and commonly occurs in young to middle-aged individuals (1–3). The duodenal BGH primarily consists of Brunner glands, smooth muscle, fibrous connective tissue, and blood vessels (4). It has been associated with malignant and pre-malignant lesions such as epithelial dysplasia, duodenal adenocarcinoma, and carcinoid tumors. Previous reports indicate that duodenal BGHs can cause anemia and gastrointestinal obstruction, which are often discovered during investigations for these conditions (5, 6).

Computed tomography (CT) scans reveal a polypoidal mass originating from the duodenal wall, accompanied by secondary manifestations of the hamartoma, including intestinal obstruction or intussusception. However, few reports analyzed the imaging manifestation of this hamartoma in detail.

Herein, we describe a case of a pedunculated duodenal BGH detected on contrast-enhanced computed tomography. Surgery was performed, and the diagnosis was confirmed through histopathology. This report aims to enhance our understanding of this condition.

## Case presentation

### Chief complaints

A 43-year-old man was admitted to the gastroenterology department with mild discomfort for 6 to 7 years and has recently experienced bloating without vomiting or melena.

### History of present illness

The patient presented with bloating for 6 to 7 years without manifesting any noticeable symptoms and was observed in another hospital. Upon retrospective analysis of the patient’s medical history, it was determined that the patient was admitted to the Department of Urology for treatment of nephrolithiasis in 2023. The subsequent abdominal CT scan showed similar imaging characteristics to those in the current study, and gastrointestinal endoscopy was also recommended for performance. However, the patient did not choose to undergo further examination.

### History of past illness

The patient had no known comorbidities or past illnesses.

### Personal and family history

There is no family history of polyps or tumors.

### Physical examination

The abdominal physical examination showed no tenderness.

### Laboratory examinations

This time, laboratory examinations detected fecal occult blood only weakly, prompting an endoscopic evaluation that included esophagogastroduodenoscopy.

### Imaging examinations

**Figure 1:**
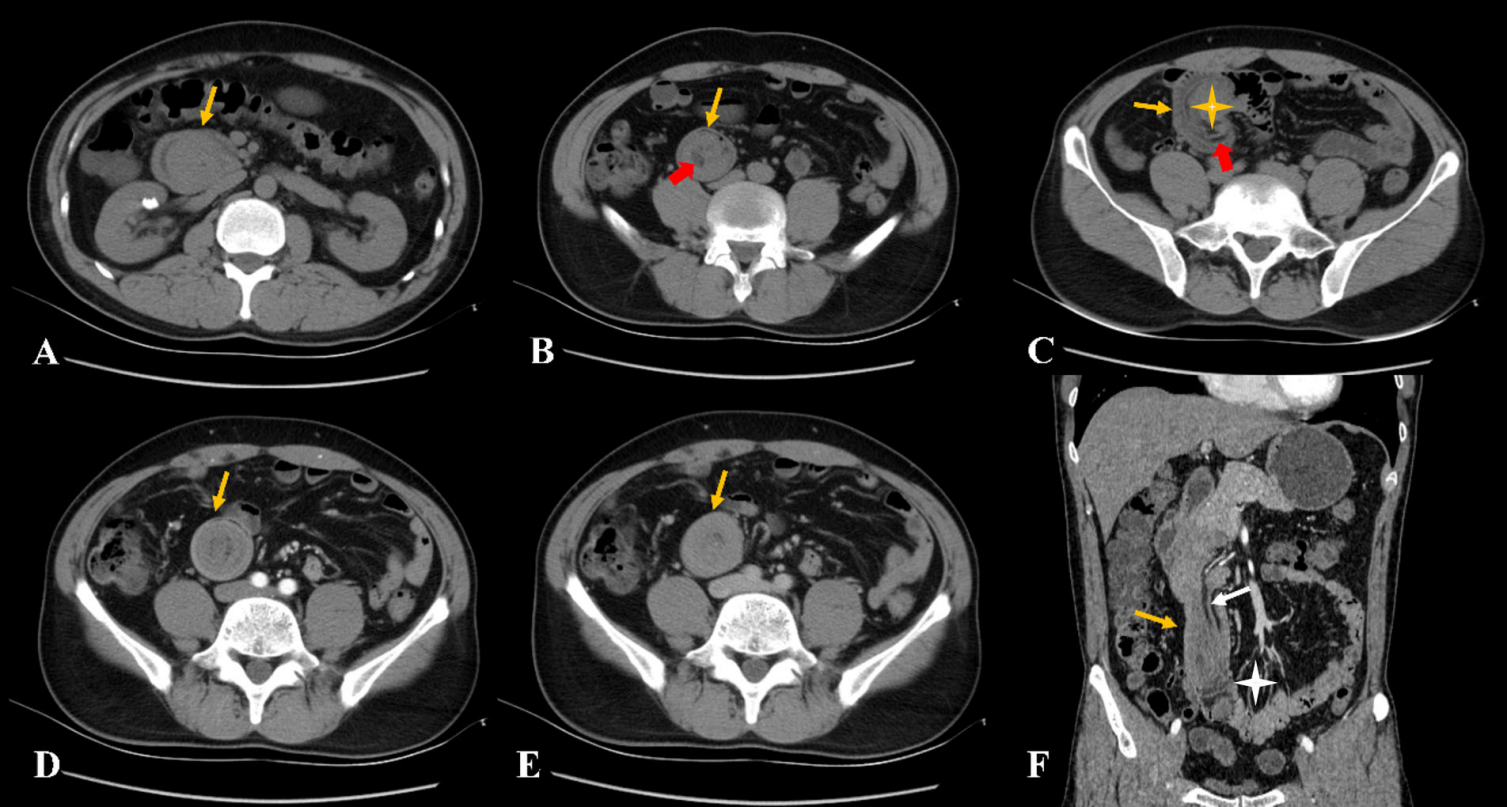
**(A–C)** The non-contrast computed tomography showed a strip-like lesion with fat attenuation (red arrow) in the duodenal intestinal canal. The lesion obstructed the descending part of the duodenum, with significant dilatation (yellow asterisk). **(D–F)** The contrast-enhanced computed tomography showed the lesion with mild to moderate enhancement with incomplete intussusception, and mesentery and mesenteric blood vessels can be observed (white arrow). The proximal small intestine was located in the pelvic cavity (white asterisk).

CT examination of the abdomen revealed marked thickening of the duodenal walls, and a strip-like, mixed-density lesion with fat attenuation was observed in the intestinal canal, measuring an average of 35 Hounsfield units (HU), which was blocking the duodenal duct. The proximal intestine was dilated. The third portion of the duodenum (transverse segment) and the proximal jejunum were located in the pelvis. Contrast-enhanced abdominal CT scans later revealed thickening of the duodenal walls with marked enhancement and a strip-like, mixed-density lesion with mild to moderate enhancement (density of 49 HU in the arterial phase and 61 HU in the venous phase). Moreover, part of the enhanced duodenal intestinal wall was stretched into the intestinal lumen. Fat attenuation was observed between the lesion and the intestinal wall, with mesentery blood vessels seen in enhancement, and the image showed incomplete intussusception (**Figure 1**). The mucosal layer of the lesion and the adjacent duodenal walls were seen unclearly on contrast-enhanced CT images.

### Final diagnosis

**Figure 2:**
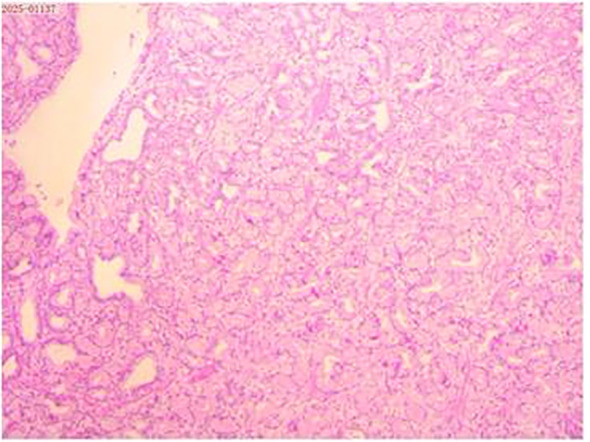
Histopathological examination of the resected specimen (hematoxylin and eosin; × 40) revealed that the mass exhibited lobulated hyperplasia of Brunner’s glands, partitioned by smooth muscle bundles, with lymphocytic aggregates.

The histological examination revealed that the mass exhibited lobulated hyperplasia of Brunner’s glands, separated by smooth muscle bundles, with interspersed adipose tissue and lymphocytic aggregates (**Figure 2**).

### Treatment

**Figure 3:**
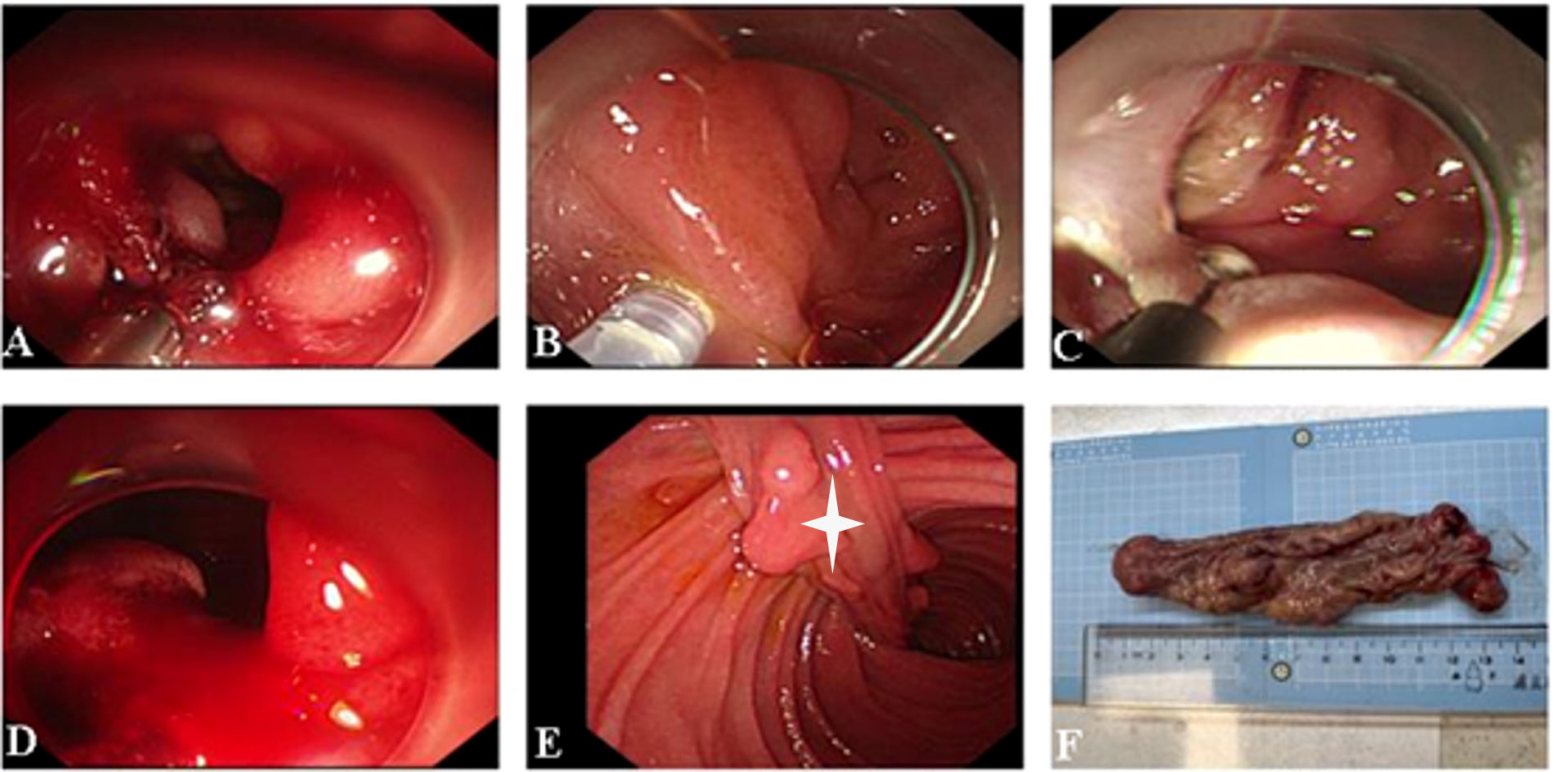
**(A–E)** Endoscopic submucosal dissection of the duodenal polyp. The stalk of the lesion is visible (white asterisk). **(F)** Macroscopic appearance of the polyp after resection.

Gastroscopy was performed, revealing a pedunculated polyp with a base visualized at the level of the descending duodenum. The polyp prolapses into the horizontal duodenum, partially obstructing the descending duodenum, and the intestinal canal was dilated. Endoscopic submucosal dissection was carried out around the root of the lesion, gradually exposing it. The adhesion between the lesion and the muscularis propria of the duodenum was evident, and the lesion was resected entirely with the endoscope—the polyp measuring 2.5 × 15.0 cm (**Figure 3**).

### Outcome and follow-up

**Figure 4:**
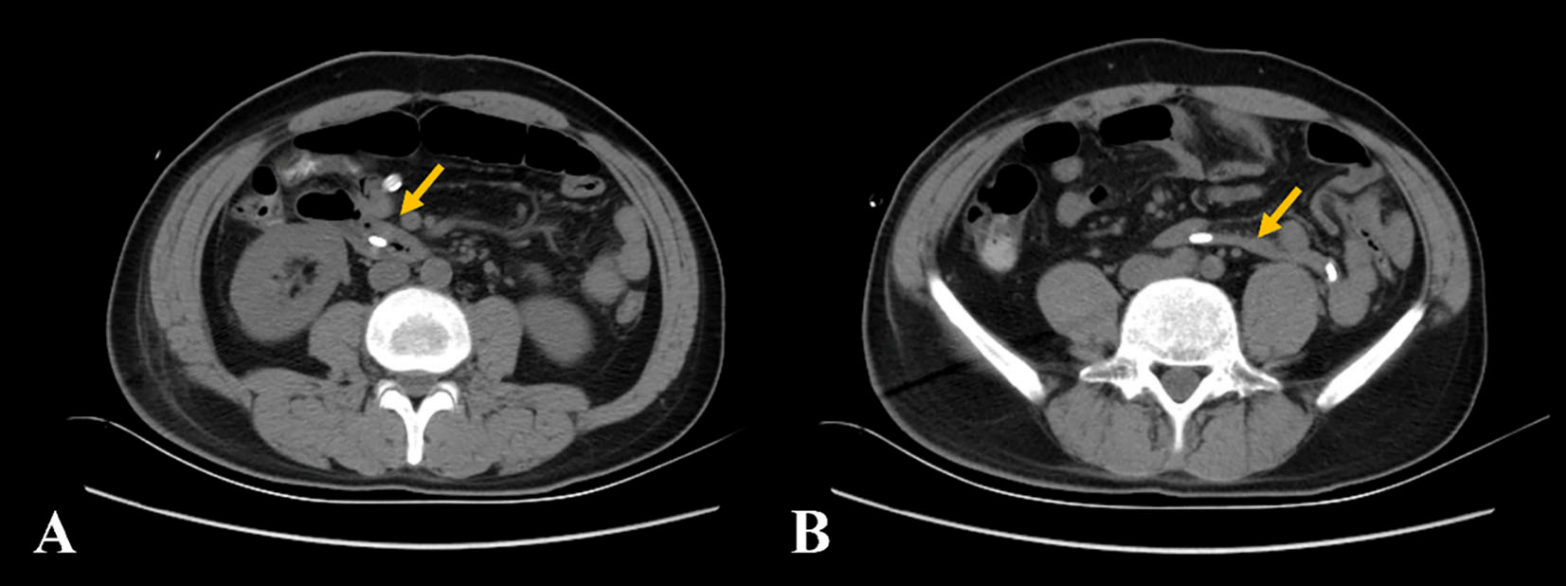
**(A, B)** The non-contrast computed tomography showed the resolution of duodenal wall abnormalities with the restoration of normal anatomical position (yellow arrow).

The patient recovered well after surgery. CT demonstrated the resolution of duodenal wall abnormalities with the restoration of normal anatomical position (**Figure 4**). The current basic situation is good.

## Discussion

The duodenal BGH, also known as BGA, is a solitary hamartomatous polyp that is typically benign and commonly occurs in young and middle-aged individuals (5, 6). Its formation arises from variations in the proportion, distribution, and differentiation of various tissue components compared with normal tissues. The duodenal BGH primarily consists of Brunner glands, smooth muscle, fibrous connective tissue, and fat components (7). Few cases have reported the immunohistochemical results of this hamartoma. In the report by Alsugair et al. (4), it was mentioned that the smooth muscle fibers separated the Brunner gland lobules (AE1/AE3). Brunner glands express MUC6 and also express MUC5AC focally. The Brunner gland did not express MUC2.

The Brunner’s gland is situated in the submucosal layer and the muscularis mucosa. The abnormal proliferation of Brunner’s gland is classified into three subtypes: diffuse hyperplasia, circumscribed hyperplasia, and glandular adenoma (8, 9). Moreover, glandular adenoma has been modified as BGA. The number and size of the Brunner glands decrease distally, resulting in a lower incidence of the hamartomas (10). Therefore, it is currently believed that duodenal BGH mainly occurs in the duodenal bulb, followed by the descending and horizontal sections of the duodenum. The polyps were rarely found in the jejunum. The tumor is small and typically does not present obvious clinical symptoms; however, as the hamartoma grows, some case reports indicate that patients may be admitted to the hospital with gastrointestinal hemorrhage, intestinal obstruction, or intussusception secondary to the hamartoma (11, 12). The clinical manifestations are usually atypical. Most patients show normal results in biochemistry examinations.

The discovery and diagnosis of duodenal BGHs may require imaging assistance. The hamartomas can be identified through hypotonic double-contrast barium radiography as well-circumscribed, rounded filling defects in the duodenal bulb or descending segment of the duodenum.

CT exhibits sensitivity in detecting hamartomas and secondary complications associated with these lesions. Due to the small number of cases, few specific imaging findings were found. In many instances (4, 8, 9, 13, 14), the hamartomas were located at the duodenal bulb or the descending sections of the duodenum, and the enhancement of CT was heterogeneous. CT showed only marked thickening of the gastrointestinal wall when the hamartomas were small, and hamartomas that appeared as polypoid masses protruding into the lumen, mostly with proximal intestinal dilation or different degrees of intussusception when the hamartomas were larger. Because the Brunner gland is located in the submucosa, most duodenal BGHs can be covered by normal intestinal mucosa. Contrast-enhanced CT scans showed that the lesions were located within the intestinal cavity, and the mucosal layer was visible on the CT images. The coronal section revealed multiple layers of mucosa in the intestinal cavity, resembling “intussusception,” which may lead to misdiagnosis of intussusception on imaging (4, 8, 9, 13, 14). In this case, perhaps due to the large polyp, the mucosal layer of the hamartoma was unclear in relation to the mucosal layer of the intestinal wall, and the “illusion of intestinal intussusception” was not evident. However, the adjacent intestinal wall was stretched into the lumen of the intestine. The stretched intestinal wall showed mucosal enhancement in the contrast-enhanced CT. The mesentery and mesenteric blood vessels can be seen between the lesion and the intestinal wall, suggesting that this case was likely a combination of incomplete intussusception, rather than pseudo-intussusception caused by multiple mucosal folded between the lesion and the intestinal wall. It is necessary to identify whether there is a true intussusception on the image.

However, the hamartoma exhibited heterogeneous density on CT imaging, with fat attenuation within the lesion. It was relatively rare in previous reports (4, 8, 9, 13, 14). The duodenal BGH can contain mature adipocytes, and fat density can be seen in the tumor on CT imaging. Therefore, we hypothesized that the imaging finding of an elongated intraluminal mass lesion with heterogeneous density and fat attenuation in the duodenum should raise clinical suspicion for a pedunculated duodenal BGH, especially when accompanied by intussusception or obstruction.

The duodenal BGHs should also be differentiated from Peutz–Jeghers polyps (5). Peutz–Jeghers syndrome (PJS) is an autosomal dominant genetic disorder that can be characterized by pigmentation of the skin and mucosa, as well as multiple polyps in the gastrointestinal tract. Peutz–Jeghers polyp has a high risk of malignant transformation, mostly occurring in the duodenum and proximal jejunum. The number of polyps increases with age, often presenting as multiple pedicled polyps, and over 90% of polyps are misfit polyps with a dendritic shape (15). In contrast, primary duodenal neoplasms such as gastrointestinal stromal tumors (GISTs), neuroendocrine neoplasms (NENs), and other malignancies typically present as well-defined soft tissue masses (16). Brunner’s hamartomas need to be differentiated from the following lesions: ① GISTs: The most common site of occurrence is the stomach, followed by the small intestine; they can occur in any part of the duodenum but are most common in the descending part of the duodenum. Located in the submucosa, GISTs can present as intraluminal or exophytic lesions, showing a spherical or spheroidal shape, but usually without a pedicle. GISTs do not contain fat components, and common manifestations include cystic degeneration, hemorrhage, and calcification; they show noticeable and persistent enhancement on enhanced CT examination. ② NENs: Most neuroendocrine neoplasms are round nodules or masses, and some cases are accompanied by gastrointestinal ulcers. Most neuroendocrine neoplasms show noticeable enhancement, and some may have necrosis and cystic degeneration (17, 18). ③ Adenocarcinomas: They can occur in any part of the duodenum. On CT, they present as thickened intestinal walls or masses protruding into the intestinal lumen, with irregular margins, and may be accompanied by ulcers. The degree of enhancement is generally obvious and heterogeneous, with no fat components inside. The intestinal wall of the lesioned segment is thickened and stiff, which may be accompanied by perienteric fat invasion and lymph node metastasis; some patients have elevated carcinoembryonic antigen (CEA) levels. Therefore, the absence of intralesional fat-density components in these entities might serve as the key feature for differentiation. In addition, combined with contrast-enhanced CT protocols, this modality can differentiate BGHs from other intestinal neoplasms.

However, whether this finding can be considered a specific imaging feature for this type of hamartoma still requires further corroboration through the analysis of a larger case series. Magnetic resonance enterography (MRE) can provide images and diagnostic results similar to those from CT. In some reports, magnetic resonance imaging (MRI) can be used to regularly monitor the presence of hamartomas in the bowel (15).

Although duodenal BGHs primarily appear as benign lesions, their potential for malignant transformation requires clinical vigilance. Current guidelines recommend proactive surgical intervention for lesions larger than 1 cm in diameter (19). The primary therapeutic methods include endoscopic resection for accessible lesions and surgical duodenectomy for more complex cases. Evidence from longitudinal studies indicates the need for prolonged clinical surveillance for all diagnosed patients due to the documented risk of occult malignancy in these proliferations (2, 20).

## Conclusion

Duodenal BGHs are typically benign, characterized by lobulated hyperplasia of the Brunner gland associated with the presence of other mature tissues. The hamartomas may present clinically with upper gastrointestinal hemorrhage and sometimes intestinal obstruction or intussusception. Most BGHs are located in the submucosa at the duodenal bulb or the descending sections of the duodenum, with a narrow peduncle in morphology, and appear heterogeneous on contrast-enhanced CT. The imaging finding of an elongated intraluminal mass lesion with heterogeneous density and fat attenuation may raise clinical suspicion for a pedunculated duodenal BGH, assisting in differential diagnosis.

## Data Availability

The original contributions presented in the study are included in the article/supplementary material. Further inquiries can be directed to the corresponding author.
